# Interpretable Recognition for Dementia Using Brain Images

**DOI:** 10.3389/fnins.2021.748689

**Published:** 2021-09-24

**Authors:** Xinjian Song, Feng Gu, Xiude Wang, Songhua Ma, Li Wang

**Affiliations:** ^1^Department of Rehabilitation Medicine, Affiliated Nantong Rehabilitation Hospital of Nantong University, Nantong, China; ^2^Department of Medical Image, Affiliated Nantong Rehabilitation Hospital of Nantong University, Nantong, China; ^3^Department of Neurology, Affiliated Nantong Rehabilitation Hospital of Nantong University, Nantong, China; ^4^School of Information Science and Technology, Nantong University, Nantong, China; ^5^Research Center for Intelligence Information Technology, Nantong University, Nantong, China; ^6^Nantong Research Institute for Advanced Communication Technologies, Nantong, China

**Keywords:** dementia, Alzheimer’s disease, brain images, TSK fuzzy systems, interpretability

## Abstract

Machine learning-based models are widely used for neuroimage-based dementia recognition and achieve great success. However, most models omit the interpretability that is a very important factor regarding the confidence of a model. Takagi–Sugeno–Kang (TSK) fuzzy classifiers as the high interpretability and promising classification performance have widely used in many scenarios. TSK fuzzy classifier can generate interpretable fuzzy rules showing the reasoning process. However, when facing high-dimensional data, the antecedent become complex which may reduce the interpretability. In this study, to keep the antecedent of fuzzy rule concise, we introduce the subspace clustering technique and use it for antecedent learning. Experimental results show that the used model can generate promising recognition performance as well as concise fuzzy rules.

## Introduction

Dementia is a clinical syndrome with progressive cognitive decline. The number of patients suffering from dementia worldwide is as high as 47.5 million. With the aging of the population, it is estimated that the number of people will be 75 million in another 20 years, and this number will triple in the next 50 years (Chen and Herskovits, 2010; Bansal et al., 2018). Alzheimer’s Disease (AD) is the most common cause of dementia, which has a long incubation period and prodromal stage, and the average clinical treatment time is 8–10 years (Moradi et al., 2015; Zhang et al., 2015; Liu et al., 2020). There is currently no treatment that can stop, delay or reverse the progression of the course of AD. Neuropathological studies have found that the main causes of AD are the accumulation of amyloid plaques outside the cell, the tangling of neuronal fibers within the cell, the deterioration of synapses, and the death of neurons. The aggregation of amyloid plaques interferes with synaptic activity and brings about a series of inter-neural and intra-neuronal effects, and ultimately leads to the death of brain cells.

The current three-dimensional medical imaging technology is becoming more and more mature. Obtaining multiple modal medical images for each patient has become a diagnostic trend of AD. Such as complex but non-invasive magnetic resonance imaging (MRI) and positron emission tomography (PET) can realize the diagnosis of the disease and monitor its progress and the effect of subsequent treatment (Mirzaei et al., 2016; Zhang et al., 2021b). MRI is one of the neuroimaging modalities with high resolution imaging and high brain tissue contrast. It can well quantify the brain tissue atrophy in patients with AD and mild cognitive impairment (MCI). PET is another neuroimaging modality for detecting AD. AD and MCI patients usually reduce glucose metabolism in certain areas before the brain is significantly atrophy. PET can monitor changes in glucose metabolism in the human body. In reality, the diagnosis of AD and MCI is still based on doctor’s clinical diagnosis and psychometric evaluation. This method greatly wastes manpower and material resources, and at the same time produces highly subjective judgment results, which can easily lead to misdiagnosis and missed diagnosis. Patients with MCI will experience slight memory loss, but this will not have a substantial impact on the life of the patient. Therefore, the cognitive level of early MCI may not be judged according to the evaluation of the medical diagnosis cognitive scale. If you ignore it, then the risk of conversion to AD is extremely high, resulting in irreversible consequences, which is extremely detrimental to the early prevention of AD and MCI. Therefore, when looking for effective treatments to prevent or slow down the progress of AD, it is necessary to better develop medical auxiliary diagnostic tools, and the development of these tools also helps to measure the efficacy of new therapies.

Using machine learning methods to classify is to automatically learn the existing data, then obtain the corresponding patterns. Using such patterns, a set of unknown input samples can be judged to achieve classification and prediction. Machine learning methods have been widely used in character recognition, face recognition, speech recognition, and medical classification. Based on MRI, Cuingnet et al. (2011) compared 10 different AD automatic classification methods and compared the difference between extracting features of the whole brain and features of some related regions. The experiment proved that the effect of selecting a group of related regions is better than selecting the whole brain. Area or separate hippocampus area. Querbes et al. (2009) used MRI images to measure the thickness of the cerebral cortex as a classification feature. The thickness of the cortex can characterize brain atrophy and achieved 85% classification accuracy in the classification of AD and HC. Wen et al. (2008) used principal component analysis to make feature selection for PET features, and then used logistic regression to classify AD and healthy controls (HC) and achieved a classification accuracy of 82%. Zhang et al. (2011) used support vector machine (SVM) to classify AD and HC based on multi-modal features and achieved a classification accuracy of 93.2%. Tong et al. (2017) used four modal features, namely MRI, PET, cerebrospinal fluid (CSF) and genetic information, and used an unsupervised metric fusion method based on cross-diffusion to perform feature fusion, and then classification of AD, MCI, and HC by Random Forest. The classification accuracy of AD and HC is 91.8%, and the classification of MCI and HC is 79.5%.

Although machine learning-based methods have been achieved great successful in recognition for dementia caused by AD, an important issue current models do not consider is the interpretability of a model. The interpretability of a model means that the model is not a black box, it has a mechanism to tell users how it works. Takagi–Sugeno–Kang (TSK) fuzzy classifiers as the high interpretability and promising classification performance have widely used in many scenarios (Visalakshi and Radha, 2014; Zhang et al., 2017; Jiang et al., 2020a; Xia et al., 2020). Compared with SVM (Zhang et al., 2021a), neural networks (NN), Random Forest, etc., TSK fuzzy classifiers are rule-based, and they can generate interpretable fuzzy rules which provide the evidence for the final classification results. However, TSK fuzzy classifiers are easy to suffer from “rule explosion” in the high-dimensional feature space. What’s more, the high-dimensional feature space also leads to very complicated antecedents of fuzzy rules. Therefore, during the training phase, how to reduce irrelevant features is very important. To this end, in this study, we introduce a subspace clustering technique to the antecedent learning phase to ensure a concise antecedent of each fuzzy rule. The contributions of this study are summarized as follows.

(i) In order to keep the antecedents of fuzzy rules concise, a subspace clustering technique is introduced to reduce irrelevant features during antecedent learning.

(ii) We conduct extensive experiments to demonstrate the promising performance and good interpretability of our method.

## Data and Methods

### Data

**FIGURE 1:**
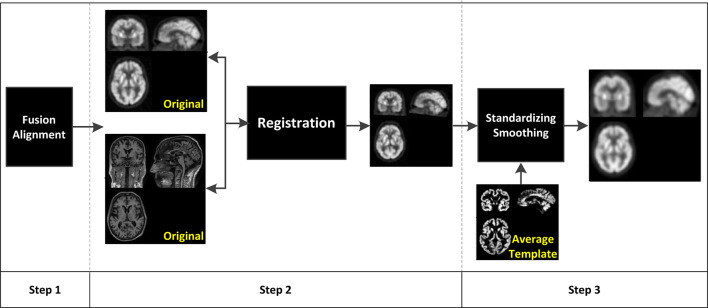
Data preprocessing pipeline of positron emission tomography (PET) images.

In this study, our brain PET images are provided by the Alzheimer’s Disease Neuroimaging Initiative (ADNI) which is a 5-year public partnership sponsored by several institutes, companies, and non-profit organizations (Zhang et al., 2021b). Figure 1 illustrates the data preprocessing pipeline of PET images, which can be divided into three main steps. In the first step, each subject in ADNI contains 96 PET images. Statistical parametric mapping (SPM) (Muzik et al., 2000) is used to fuse these PET images to construct a 3-D one which has brain spatial information and the feature information between tissue structures are also retained. In addition, motion correction is performed due to head motion. In the second step, the MRI image and PET image of each subject are registered, and affinely aligned. In the third step, the average template data generated in Figure 1 is used to spatially normalize all PET images to the standard MNI space. PET images are also smoothed (8 mm Gaussian) to avoid the influences caused by noises.

The automated anatomical atlas (AAL; Rolls et al., 2020) which is available as a toolbox^1^ for SPM is used as a template to extract original features from PET images. Based on AAL, the brain is segmented into 116 regions, and we select 90 regions from the cerebrum for feature extraction. To be specific, firstly, the PET images are resampled to the same size as the AAL template so that each region is in correspondence spatially. The size of AAL template is 61 × 73 × 61. Then we extract average intensity values from all regions of PET images as original features for our proposed classification model.

### Methods

**FIGURE 2:**
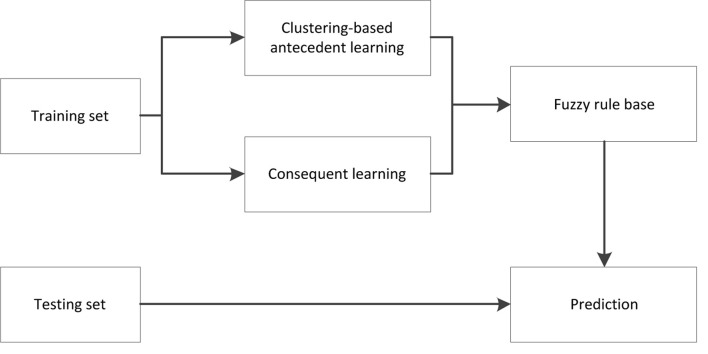
Learning framework of Takagi–Sugeno–Kang (TSK) fuzzy classifiers.

Figure 2 illustrates the learning framework of our TSK fuzzy classifier. The training contains two separate sections, clustering-based antecedent learning and consequent learning. In the following, we will focus on subspace clustering-based antecedent learning.

#### Notations

In this study, **X** = [**x**_1_,**x**_2_,…,**x**_*n*_] ∈ *R*^*N*×*d*^ is used to represent the training sample set and y = [*y*_1_,*y*_2_,…,*y*_*n*_]^T^ ∈ *R*^*n*×1^ is the corresponding label vector. An arbitrary sample **x**_*i*_ can be denoted as [*x*_*i*1_,*x*_*i*2_,…,*x*_*id*_]^T^. For an arbitrary matrix **B**, we use *b*_*ij*_ to represent its element in the *i*-th row and *j*-th column and **b**_*i*_ to represent its *i*-th row.

#### Subspace Clustering-Based Takagi–Sugeno–Kang Fuzzy System

In this section, we develop a TSK fuzzy classifier to recognize AD patients. TSK fuzzy classifiers are rule-based models, the *k*-th fuzzy rule can be expressed as follows,


(1) $$\text{If}\,\,x_{i\,1}\,\text{is}\,A_{1}^{k}\,\wedge \,x_{i\,2}\,i\,s\,A_{2}^{k}\,\wedge \,\ldots \,\wedge \,x_{i\,d}\,\text{is}\,A_{d}^{k},\text{then}\,\,f^{k}\,\left({\text{x}}_{i}\right)=p_{0}^{k}+p_{1}^{k}\,x_{i\,1}+\cdots +p_{d}^{k}\,x_{i\,d}$$

where $A_{i}^{k}$ denotes the fuzzy subset regarding the *i*-th feature, $\left[p_{0}^{k},p_{1}^{k},\ldots ,p_{d}^{k}\right]$ denotes the consequent parameter, *f^k^*(**x**_*i*_) denotes the output of the *k*-th fuzzy rule regarding **x**_*i*_. When we adopt multiplication as conjunction and implication, addition as combination, and the center of gravity as defuzzification, the output of the TSK fuzzy classifier can be expressed as follows,


(2) y⁢(xi)=∑k=1Kμk⁢(xi)∑k=′1Kμk′⁢(xi)⁢fk⁢(xi)=∑k=1Kμ~k⁢(xi)⁢fk⁢(xi)

where *K* denotes the number of fuzzy rules, μ^*k*^(**x**_*i*_) and ${\tilde{\text{μ}}}^{k}\,\left({\text{x}}_{i}\right)$ are usually called as the firing strength and the normalized firing strength, respectively, which are defined as follows,


(3) $${\text{μ}}^{k}\,\left({\text{x}}_{i}\right)=\prod_{j=1}^{d}{\text{μ}}_{A_{i}^{k}}\,\left({\text{x}}_{i}\right)$$

(4) μ~k⁢(xi)=μk⁢(xi)∑k=′1Kμk′⁢(xi)

where ${\text{μ}}_{A_{i}^{k}}\,\left({\text{x}}_{i}\right)$ denotes the membership function the fuzzy subset $A_{i}^{k}$. In this study, we adopt the Gaussian function as the membership function, which is defined as follows,


(5) $$\mu_{A_{i}^{k}}\,\left({\text{x}}_{i}\right)=\text{exp}\,\left(\frac{-\left({\text{x}}_{i}-{\text{v}}_{i}^{k}\right)}{2\,\sigma_{i}^{k}}\right)$$

where ${\text{v}}_{i}^{k}$ and $\sigma_{i}^{k}$ are the antecedent parameters.

Once the antecedent parameters are determined clustering techniques or other schemas, let


(6) $${\text{x}}_{e}={\left(1,{\text{x}}_{i}^{T}\right)}^{T}$$

(7) $${\tilde{\text{x}}}^{k}={\tilde{\mu }}^{k}\,\left({\text{x}}_{i}\right)\,{\text{x}}_{e}$$

(8) $${\text{x}}_{g}={\left({\left({\tilde{\text{x}}}^{1}\right)}^{T},{\left({\tilde{\text{x}}}^{2}\right)}^{T},\ldots ,{\left({\tilde{\text{x}}}^{K}\right)}^{T}\right)}^{T}$$

(9) $${\text{p}}^{k}={\left({\text{p}}_{0}^{k},{\text{p}}_{1}^{k},\ldots ,{\text{p}}_{d}^{k}\right)}^{T}$$

(10) $${\text{p}}_{g}={\left({\left({\text{p}}^{1}\right)}^{T},{\left({\text{p}}^{2}\right)}^{T},\ldots ,{\left({\text{p}}^{K}\right)}^{T}\right)}^{T}$$

Based on (6)–(10), we can update the output of the TSK fuzzy classifier as follows,


(11) $$y\,\left({\text{x}}_{i}\right)={\text{p}}_{g}^{T}\,{\text{x}}_{g}$$

In general, the optimization of the TSK fuzzy classifier can be conduct separately. As for the antecedent, clustering is usually used, and for the consequent, we see from (11) that it can be solved by many techniques because it can be considered as a linear regression model. As we stated before that the number of features involved in antecedents of fuzzy rules is a key factor to the interpretability of TSK fuzzy systems. Therefore, to reduce irrelevant features and make the antecedents of fuzzy rules more concise, in our study, we introduce a subspace clustering technique to optimize the antecedent. The core idea is that it uses a weight matrix to measure the weights of features in each cluster. The objective function of the introduced clustering technique is formulated as follows,


(12) $$J\,\left(U,V,W\right)=\sum_{c=1}^{C}\sum_{i=1}^{N}\mu_{c\,i}^{m}\,\sum_{j=1}^{d}w_{c\,j}\,{\left(x_{i\,j}-v_{c\,j}\right)}^{2}+\sum_{c=1}^{c}\delta_{c}\,\sum_{j=1}^{d}w_{c\,j}^{2},$$

(13) $$s.t.\sum_{c=1}^{C}\mu_{c\,i}=1,\sum_{j=1}^{d}w_{c\,j}=1$$

where *μ*_*ci*_ is an element of **U** which denotes the fuzzy membership degree of sample **x**_*i*_ belonging to cluster *c*, *v*_*cj*_ is an element of **V** which denotes the *j*-th feature of the *c*-th cluster’s center, and *w*_*cj*_ is an element of **W** which denotes the weight of the *j*-th feature in the *c*-th cluster. δ_*c*_ is constant of the *c*-th cluster, *C* denotes the number of clusters, *N* denotes the number of training samples, *d* denotes the number of features and *m* denotes the fuzzy exponential.

According to Frigui and Nasraoui (2004), by introducing Lagrangian multipliers, we have several updating rules as follows,


(14) $$w_{c\,j}=\frac{1}{d}+\frac{1}{2\,\delta_{c}}\,\sum_{i=1}^{N}\mu_{c\,i}^{m}\,\left(\frac{{||x_{i}-v_{c}||}^{2}}{d}-{\left(x_{i\,j}-v_{c\,j}\right)}^{2}\right),$$

(15) $$\delta_{c}=\frac{\sum_{i=1}^{N}{\text{μ}}_{c\,i}^{m}\,\sum_{j=1}^{d}w_{c\,j}\,{\left({\text{x}}_{i\,j}-{\text{v}}_{c\,j}\right)}^{2}}{\sum_{j=1}^{d}w_{c\,j}^{2}}$$

(16) μc⁢i=1∑c=′1C[∑j=1dwc⁢j⁢(xi⁢j-vc⁢j)2∑j=1dwc⁢j′⁢(xi⁢j-vc⁢j′)2]1/(m-1)

(17) $$v_{c\,j}=\frac{w_{c\,j}\,\sum_{i=1}^{N}{\text{μ}}_{c\,i}^{m}\,x_{i\,j}}{w_{c\,j}\,\sum_{i=1}^{N}{\text{μ}}_{c\,i}^{m}}$$

When the subspace clustering converges, we can use the following equations to calculate the antecedent parameters ${\text{V}}_{i}^{k}$ and $\sigma_{i}^{k}$,


(18) $${\text{v}}_{i}^{k}=\frac{\sum_{i=1}^{N}{\text{μ}}_{k\,i}\,{\text{x}}_{i\,j}}{\sum_{i=1}^{N}{\text{μ}}_{k\,i}},$$

(19) $$\sigma_{i}^{k}=\frac{h\,\sum_{i=1}^{N}{\text{μ}}_{k\,i}\,{\left({\text{x}}_{i\,j}-{\text{v}}_{i}^{k}\right)}^{2}}{\sum_{i=1}^{N}{\text{μ}}_{k\,i}},$$

where *h* is a user-defined parameter. Based on the subspace clustering technique, the training algorithm of the TSK fuzzy classifier is listed as follows. Notably, the stopping threshold ε is set to 1e-5. Detailed algorithm steps are shown in Algorithm 1.

**Algorithm 1:** Subspace clustering-based TSK fuzzy system.

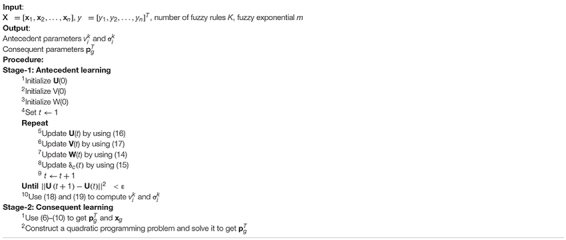

## Results

### Setups

In our experiments, the fuzzy exponential *m* is set to 2, the number of fuzzy rules is set to 15, *h* in (19) is set to 0.5. The original number of features we obtained via the pipeline in Figure 1 is 93. We use the feature selection method proposed in Jiang et al. (2020b) to reduce the dimension to 15.

To highlight the interpretability and performance of the subspace-based TSK fuzzy classifier, we introduce the classical one order TSK fuzzy classifier (1-TSK-FC) (Jiang et al., 2016) for comparison.

We introduce accuracy (ACC) and model complexity (MC) to evaluate the performance and interpretability, where ACC is defined as the ratio of correctly classified samples to the total number of samples, and MC is defined as the number of parameters participating the training phase.

### Experimental Results

**FIGURE 3:**
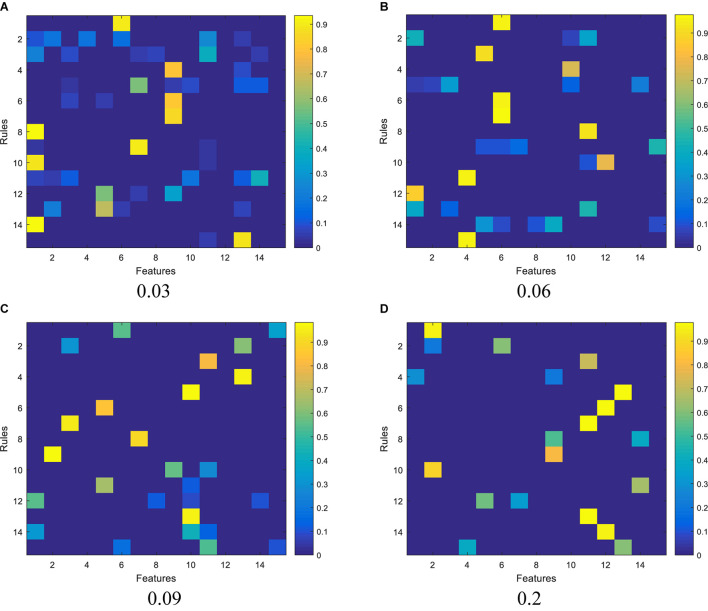
Activation of features by the subspace clustering under different thresholds: **(A)** 0.03, **(B)** 0.06, **(C)** 0.09, and **(D)** 0.2.

We report the experimental results from 3 aspects. The first one is the feature activation results, as shown in Figure 3, regarding the subspace clustering for antecedent learning. In Figure 3, each subpanel represents the activated features for each fuzzy rule under different thresholds, the brighter the color, the greater the corresponding weight of each feature in each fuzzy rule. It observes that as the threshold increases, the number of activated features contained in each rule begins to decrease.

**FIGURE 4:**
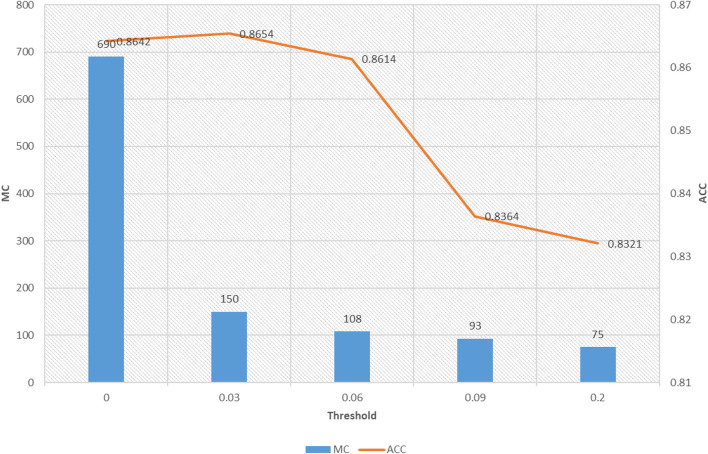
Model complexity and accuracy under different thresholds.

The second one is the relationship between model complexity and accuracy, which is illustrated in Figure 4. As we stated before, model complexity can be quantificationally measured by the involved number of parameters during antecedent learning and consequent learning. For example, when the threshold is set to 0.06, based on the feature reduction result shown in Figure 3B, the number of features involved in each feature is 1, 3, 1, 1, 5, 1, 1, 1, 4, 2, 1, 1, 3, 5, and 1, respectively. According to (5), we know that each feature needs two parameters, so, during the antecedent learning phase, the number of parameters each feature needs is 2, 6, 2, 2, 10, 2, 2, 2, 8, 4, 2, 2, 6, 10, and 2, respectively. During the phase of consequent learning, according to (1), we know that each feature needs *d* + 1 parameters, where *d* is the current dimension after feature reduction. That is, each feature needs 2, 4, 2, 2, 6, 2, 2, 2, 5, 3, 2, 2, 4, 6, and 2 parameters, respectively. Therefore, model complexity under threshold being 0.06 is 108. When the threshold is set to 0, it means that the classifier degenerates into 1-TSK-FS. From Figure 4, it observes that model complexity of 1-TSK-FS is 690, which is seriously higher than that of subspace clustering-based learning. What is more, the classification performance does not reduce significantly with the decreasing of model complexity. For example, when the model complexity is 75, the corresponding performance still keeps in a reasonable level.

**FIGURE 5:**
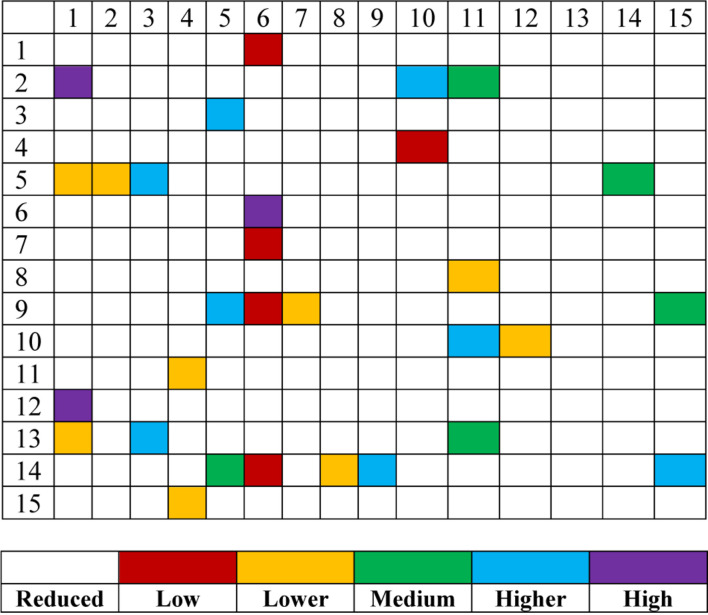
Linguistic meaning of activated features of each rule.

The third one is the results of model interpretability. In Figure 5, we assign linguistic terms “*Low*, *Lower*, *Medium*, *Higher*, and *High*” to each feature according to the antecedent parameters. Based on this assignment and the consequent parameters, Table 1 shows the rule base consisting of 15 fuzzy rules. It is easy to find that the antecedent of each fuzzy rule is very concise. Please note that the assignment of linguistic terms is based on the knowledge of expert. Different experts from different domain may have different assignment.

**TABLE 1:** Rule base.

**Fuzzy rule:** $\mathrm{If}\,x_{i\,1}\,\text{is}\,A_{1}^{k}\,\wedge \,x_{i\,2}\,\text{is}\,A_{2}^{k}\,\wedge \,\ldots \,\wedge \,x_{i\,d}\,\text{is}\,A_{d}^{k},\text{then}\,\,f^{k}\,\left({\text{x}}_{i}\right)=p_{0}^{k}+p_{1}^{k}\,x_{i\,1}+\cdots +p_{d}^{k}\,x_{i\,d}$		
**Rule No.**	**Antecedent**	**Consequent $\left[p_{0}^{k},p_{1}^{k},\ldots ,p_{d}^{k}\right]$**

1	**If** *x*_*i*6_is *Low*	[0.0558, −0.1106, −0.1158, 0.0490, −0.0513, 0.0706, 0.0274, −0.0270, 0.1006, 0.0865, 0.0762, 0.0642, 0.0642, −0.0763, 0.2249, −0.1551]

2	**If** *x*_*i*1_is *High* ∧*x*_*i*10_is *Higher*∧*x*_*i*11_is *Medium*	[−0.1494, 0.0843, −0.1473, −0.2774, 0.1236, −0.1115, 0.0795, 0.0231, −0.0210, 0.0027, 0.0950, 0.0711, 0.0687, 0.0585, −0.0767, 0.2036]

3	**If** *x*_*i*1_is*High*∧*x*_*i*10_is*Higher*∧∧*x*_*i*11_is*Medium*	[0.1820, −0.1378, −0.2689, −0.2122, 0.1615, −0.0821, −0.1418, 0.0769, 0.0151, 0.2019, 0.1007, 0.0927, 0.0750, 0.0784, 0.0609, −0.0758]

4	**If** *x*_*i*5_is*Higher*	[−0.0708, 0.1903, −0.0854, −0.1610, 0.0968, −0.0573, −0.1169, −0.1359, 0.0722, −0.0805, −0.0210, 0.0912, 0.0944, 0.0584, 0.0850, 0.0562]

5	**If** *x*_*i*10_is *Lower*	[0.0533, −0.0488, −0.0385, −0.1871, 0.0617, −0.1196, −0.1453, −0.1122, −0.1232, 0.0722, 0.0151, −0.0153, 0.0985, 0.1007, 0.0562, 0.0723]

6	**If** *x*_*i*6_is *High*	[0.0607, 0.0716, 0.2803, 0.4842, −0.1474, 0.1020, −0.1651, −0.1353, −0.1002, −0.0581, 0.0722, 0.0122, −0.0199, 0.0505, 0.1021, 0.0601]

7	**If** *x*_*i*6_is*Low*	[0.0655, 0.0750, 0.0382, −0.0501, 0.1809, −0.1283, 0.0952, −0.1584, −0.1041, 0.1218, −0.1232, 0.0708, 0.0144, 0.0629, 0.0448, 0.0987]

8	**If** *x*_*i*11_is*Lower*	[0.0922, 0.0798, 0.0277, 0.0025, −0.0677, 0.2082, −0.1321, 0.0787, −0.1413, 0.2391, −0.1002, −0.1169, 0.0718, 0.0002, 0.0808, 0.0564]

9	**If** *x*_*i*5_is *Higher* ∧*x*_*i*6_is*Low*∧*x*_*i*7_is*Lower* ∧*x*_*i*15_is *Medium*	[0.0776, 0.0968, 0.1594, 0.0372, 0.0549, −0.0278, 0.2004, −0.1404, −0.0509, 0.0285, −0.1043, −0.0917, −0.1218, 0.0821, −0.0053, 0.0459]

10	**If** *x*_*i*11_is *Higher*∧*x*_*i*12_is *Lower*	[−0.0004, 0.1066, 0.5270, −0.2723, 0.0613, 0.0965, −0.0364, 0.1864, −0.1640, 0.0788, −0.1414, −0.0815, −0.0985, −0.0874, 0.0844, 0.0041]

11	**If***x*_*i*4_is *Lower*	[0.0096, −0.0235, 0.1841, −0.0057, 0.0676, 0.0958, 0.0857, −0.0543, 0.3149, −0.1403, 0.0697, −0.1306, −0.0995, −0.0062, −0.0831, 0.0793]

12	**If** *x*_*i*1_is*High*	[0.0713, 0.0174, 0.0144, −0.2364, 0.0919, 0.0945, 0.0868, 0.0660, −0.0819, 0.1864, −0.1443, 0.0767, −0.1391, 0.0844, 0.0113, −0.0919]

13	**If** *x*_*i*1_*isLower*∧*x*_*i*3_is *Higher*∧*x*_*i*1_*isMedium*	[−0.1075, 0.0735, −0.2035, 0.2646, 0.0833, 0.1043, 0.0883, 0.0702, 0.0704, −0.0543, 0.1821, −0.1413, 0.0245, −0.0569, 0.1103, −0.0234]

14	**If** *x*_*i*5_is *Medium*∧*x*_*i*6_is *Low* ∧*x*_*i*8_is *Lower*∧*x*_*i*9_is *Higher*∧*x*_*i*15_is*Higher*	[−0.0733, −0.1272, −0.2476, −0.3230, −0.0079, 0.1286, 0.1012, 0.0762, 0.1231, 0.0661, −0.0622, 0.1852, −0.1563, 0.0165, −0.0448, 0.0573]

15	**If** *x*_*i*5_is *Lower*	[−0.0368, −0.1045, 0.0095, −0.2519, 0.0104, −0.0287, 0.1196, 0.0949, 0.0234, 0.0702, 0.0589, −0.0562, 0.2137, −0.1574, 0.0320, −0.0693]

## Discussion

Although there have many excellent models that can be used for AD detection based on neuroimages, most of them omit the interpretability that is a very important factor regarding the confidence of a model. TSK fuzzy systems are rule-based inference models which can illustrate the reasoning process of the generated results. Therefore, owning to the high interpretability, they are widely used in many application scenarios. In this study, we introduce a subspace clustering technique and embed it into the antecedent learning phase to address the issue of rule complexity caused by the high-dimensional input feature space.

The subspace clustering technique uses a weighting strategy to measure the weight of each feature in each cluster. We know that when the clustering technique is used for antecedent learning of TSK fuzzy systems, the number of clusters is set to the number of fuzzy rules. Hence, the weight of each feature in each cluster corresponds to the compatible degree of each feature in each fuzzy rule. In this study, we define a threshold to reduce the irrelevant feature to keep the antecedent concise.

Definitely, we can use different thresholds to control the feature distribution. From Figure 3, we can find that the greater the threshold, the sparser distribution of the features in each fuzzy rule. In theory, the fewer features, the more succinct the antecedent of the rule, and therefore the stronger the interpretability of the fuzzy rule. However, too few features will affect the reasoning process and thus affect the classification accuracy. As can be seen from Figure 3 that when the threshold is set from 0.06 to 0.2, the classification performance in terms of accuracy decreases from 0.8614 to 0.8321. Therefore, the threshold should be elastically set to keep the balance between classification performance and interpretability.

Overall, from the experimental results, we find that subspace clustering-based TSK fuzzy classifiers cannot only ensure promising performance but also guarantee concise antecedents of fuzzy rules. Compared with classical clustering methods, like fuzzy c-means (FCM), our method is more flexible.

## Conclusion

In this study, we employ an interpretable model to achieve the detection of AD patients based on neuroimages. Compared with existing models, it merits lie in that it can generate fuzzy rules for reasoning. What’s more, we introduce a subspace clustering technique to keep the fuzzy rule concise. In our future work, we can design more strategies to reduce the superfluous fuzzy rules to further improve the interpretability of the model.

## Data Availability Statement

Publicly available datasets were analyzed in this study. This data can be found here: http://adni.loni.usc.edu/about/.

## Author Contributions

XS, FG, XW, and SM contributed on data preprocessing. LW contributed on coding and writing. All authors contributed to the article and approved the submitted version.

## Conflict of Interest

The authors declare that the research was conducted in the absence of any commercial or financial relationships that could be construed as a potential conflict of interest.

## Publisher’s Note

All claims expressed in this article are solely those of the authors and do not necessarily represent those of their affiliated organizations, or those of the publisher, the editors and the reviewers. Any product that may be evaluated in this article, or claim that may be made by its manufacturer, is not guaranteed or endorsed by the publisher.

## Footnotes

1
http://www.gin.cnrs.fr/AAL
